# „Die assoziierende Macht der Intelligenz“: Auf der Suche nach „intelligenten“ Formen sozialer und politischer Organisation

**DOI:** 10.1007/s00048-026-00450-y

**Published:** 2026-08-25

**Authors:** Alexej Lochmatow

**Affiliations:** Martin Buber Fellow, The Hebrew University of Jerusalem, Room 324 Mt. Scopus, 9190501 Jerusalem, Israel

**Keywords:** Intelligenz, Geschichte der Soziologie, Soziale und politische Imagination, Netzwerktheorie, Soziale Gruppierung, Intelligence, History of sociology, Social and political imaginary, Network theory, Social grouping

## Abstract

Dieser Aufsatz bringt die historische Entwicklung moderner Intelligenzdiskurse in Verbindung mit der „Soziologisierung“ der Theorien sozialer und politischer Organisation. Im Zentrum der Untersuchung stehen dabei drei deutschsprachige Diskurse über „Intelligenz“, die aus unterschiedlichen historischen Zusammenhängen stammen und die Entstehung sehr unterschiedlicher politischer Institutionen rechtfertigten: Die frühneuzeitliche Vorstellung von „Intelligenz“ als einem Netzwerk wirtschaftsrelevanter Informationen; die in der ersten Hälfte des 19. Jahrhunderts diskutierten Theorien der Intelligenz als einer zentralen Machtstruktur eines Staates; und die um 1900 etablierte Auffassung von Intelligenz als sozialer Gruppe. Auf diesen Beispielen basierend wird ein neuer Ansatz zur Analyse diverser Intelligenzdiskurse entwickelt, der die Kohärenz der historiografisch etablierten Vorstellungen von „Intelligenz“ radikal hinterfragt und sich bei der Untersuchung konkreter Anwendungsbeispiele den Fragen widmet, wie „Intelligenz“ unterschiedliche wissenschaftliche Theorien plausibilisiert, welche imaginierten politischen oder sozialen Strukturen die entsprechenden Intelligenzdiskurse beglaubigen und welchen sozialen Entitäten damit eine Agency zugesprochen wird. Auf diese Weise bietet der Aufsatz ein Forschungsprogramm, das jenseits der untersuchten Diskurse angewandt werden kann.

## Abstract (en)

This article connects the historical development of modern discourses on intelligence with the sociologisation of theories of social and political organisation. The author focuses on three German discourses of “intelligence” that emerged in different historical contexts and served to justify the creation of different political institutions: the early modern conception of “intelligence” as a network of economically relevant information; nineteenth-century theories that portrayed “intelligence” as a central power structure of the state; and the notion of intelligence, or intelligentsia, as a social group that emerged around 1900. Drawing on these examples, the article develops a new approach to analysing diverse discourses of intelligence and fundamentally challenges the coherence of the established historiography on the subject. The author addresses the following questions: How did “intelligence” render various scientific theories plausible? Which imagined political and social structures were legitimised by the corresponding discourses of intelligence? And which social actors acquired agency through references to “intelligence”?

## Einleitung

Im Jahr 1826 hielt der Arzt und Philosoph Franz von Baader anlässlich der Eröffnung der Ludwig-Maximilians-Universität München eine Rede. Für diesen Anlass wählte er das Thema „die Freiheit der Intelligenz“. Ganz im Sinne der protosoziologischen Debatten seiner Zeit (z. B. Turner 1980) beschrieb von Baader „Intelligenz“ als eine Art Kraft, die nicht nur das kollektive Bewusstsein der „Societät“ schafft, sondern auch eine „natürliche“ und „organische“ Entwicklung der sozialen und politischen Gebilde ermöglicht. Er sagte:

„Was nun vorerst die sociale oder associirende Macht und Natur der […] Intelligenz betrifft, welche eben so sehr die Societät von der Despotie einzelner Doktrinen oder Meinungen als von dieser ihrer Anarchie zu bewahren strebt, so widerspricht es […] der vernünftigen Natur des Menschen, nur Einzelner (im Denken, Wollen und Schaffen) zu seyn, und somit auch seine Ueberzeugung in sich zu verschließen, weil diese Natur den gemeinschaftlichen Verstand des Einzelnen mit allen andern seiner Gattung verlangt“ (Baader 1826: 4–5).

„Intelligenz“ konnte laut von Baader also nur aus Interaktionen zwischen verschiedenen Akteur:innen entstehen und hatte eine wichtige politische Funktion zu erfüllen, nämlich die Gewährleistung der inneren Stabilität verschiedener sozialer und politischer Körper (Baader 1826: 4, 6–7).

In seinem zentralen Werk zur Akteur-Netzwerk-Theorie schrieb Bruno Latour, dass *tracing of associations* zur wissenschaftlichen Praxis wurde, die bei aller Diversität der Ansätze zur Historisierung der „Wissenschaft vom Sozialen“ als Leitmotiv der Geschichte der Soziologie begriffen werden kann (Latour 2005: 5). Die Suche nach den Kategorien, die verschiedene, aus Interaktionen zwischen Einzelakteur:innen hervorgehende Zusammenhänge wissenschaftlich fixieren könnten, war laut Latour die epistemologische Praxis, welche die Absonderung soziologischer Fragestellungen lange Zeit vor der akademischen Etablierung der Soziologie kennzeichnete (ebd.: 1–4). Die Frage danach, wer als Akteur:in solcher sozialen Interaktionen und Assoziationen gesehen werden kann, wurde ebenfalls zu einem breiten Feld der historischen Entwicklung spezifisch „soziologischer“ Fragestellungen (z. B. Brown 2014: 223–232).

Der vorliegende Aufsatz greift diesen Hinweis auf und entwirft eine Geschichte der „Intelligenz“ als Geschichte der „Soziologisierung“ der Theorien sozialer und politischer Organisation. Die verschiedenen Intelligenzdiskurse werden dabei nicht nur als integraler Teil der entsprechenden sozialen und politischen Imagination (Taylor 2003; I. Kleeberg 2014; Lüdemann 2004; Arruda 2015), sondern auch als Instrument zur Erforschung spezifisch sozialer Zusammenhänge sowie zur Zuschreibung der sozialen und politischen Agency verschiedener Akteur:innen untersucht.

Der Fokus der wissenschaftshistorischen Forschung lag bisher hauptsächlich auf den späteren, individualisierten Konzepten der Intelligenz (z. B. Lamberti 2006; Mülberger 2020; Sternberg & Preiss 2022). In zahlreichen Publikationen wurde kritisch untersucht, inwiefern die grundlegenden Kategorien der Intelligenzmessung von Rassismus (z. B. Gould 1981; Quitzow 1990; Pearson 1991; Geisthövel 2013) und genderbezogenen Stereotypen „weiblicher“ und „männlicher“ Intelligenz (Daston 1992; Gigerenzer 2022) geprägt waren. Die wissenschaftliche Auseinandersetzung um die Erblichkeit der Intelligenz entwickelte sich zu einer von der Eugenik geprägten Debatte (z. B. Evans & Waites 1981; Mensh & H. Mensh 1991; Kyllingstad 2014). Darüber hinaus erläuterten die Forschenden, wie die Intelligenzforschung einer „wissenschaftlichen“ Festschreibung von Rassen- und Nationalcharakteren dienen konnte (z. B. Gilman 1996). Neben der Rolle, die der Intelligenzdiskurs in den meritokratischen Theorien zur Entstehung einer „natürlichen Aristokratie“ oder einer „Elite“ spielte (Carson 2007), wurde „Intelligenz“ auch als Instrument sozialer und professionaler Differenzierung ausführlich diskutiert (Schregel & Broer 2020; A. Bauer 2025: 321–328). Der Hauptschwerpunkt der wissenschaftshistorischen Forschung über die kollektiven Dimensionen der „Intelligenz“ lag bisher auf den literarischen und wissenschaftlichen Theorien der Schwarmintelligenz (Horn & Gisi 2009; Johach 2020: 332–352). Die Forschenden konzentrierten sich auf unterschiedliche Anwendungsbeispiele dieser Theorien, ohne den historischen Zusammenhang mit anderen Intelligenzdiskursen zu untersuchen.

„Intelligenz“ als soziale Gruppe beziehungsweise soziale Schicht stellt ein weiteres wichtiges Thema dar, das bisher in der wissenschaftshistorischen Forschung allerdings nur begrenzt diskutiert wurde. Der größte Teil der Literatur zu diesem Thema beschäftigt sich mit dem Konzept der Intelligenzija, das überwiegend aus osteuropäischen Kontexten bekannt ist (z. B. Janowski 2014; Sdvižkov 2006/2021; Gordin & Hall 2008; Zarycki 2008; Lochmatow 2019). In der Geschichte der deutschsprachigen Soziologie wurden dieser Begriff und die dahinterstehenden Theorien der sozialen Schichtung in verschiedenen Zusammenhängen ebenfalls ausführlich diskutiert (z. B. König 1987; Hoeges 1994; Ettrich 2007; Dahms 2018: 97–112; Loader 1997, 2022). Jedoch wird „Intelligenz“ in diesen Theorien überwiegend als Teil der „Soziologie der Intellektuellen“ begriffen, was den Zusammenhang mit früheren Intelligenzdiskursen ausblendet und den Schwerpunkt nicht auf Mechanismen sozialer Entwicklung, sondern auf die „moralischen Aufgaben“ der Intellektuellen verlagert (Neun 2022). Eine reflektierte Etymologie des Begriffs wird in diesem Zusammenhang nur selten erreicht (Müller 1971: 85–94), während die deutschsprachigen Diskurse der Intelligenz des 19. Jahrhunderts in der Historiografie nur nebenbei erwähnt werden (z. B. Koselleck 1989: 399–400).

Die Geschichte der „Soziologisierung“ der wissenschaftlichen Epistemologien gehört zu den zentralen Themen der wissenschaftshistorischen Forschung (z. B. Porter & Ross 2003; Fleck & Dayé 2020; Dörk & Link 2019). Die aktuelle Forschung hat gezeigt, dass die Fragestellungen nach den sozialen Interaktionen und den Akteur:innen solcher Interaktionen bereits in den vormodernen Diskursen sichtbar wurden (z. B. Füssel & Weller 2011; Pohlig & Schlieben 2022: 9–26). Ebenso ausführlich wurden die Zusammenhänge zwischen diversen sozialen Ordnungen und den politischen Deutungen der entstehenden Sozialwissenschaften diskutiert (z. B. Falkenhayner et al. 2015; Sheehan & Wahrman 2015; Kleeberg 2015).

Der vorliegende Aufsatz zeigt auf, dass die bisher separat voneinander untersuchten Geschichten der Sozialwissenschaften in Verbindung mit der Entwicklung diverser Intelligenzdiskurse gebracht werden können. „Intelligenz“ wird als eine Art Mediatorin und Operatorin der Anwendung verschiedener sozialer und politischer Theorien begriffen. Die begriffsgeschichtliche Gestaltung des Aufsatzes (Koselleck 2006) soll als Grundlage für neue Fragestellungen dienen. Methodologisch wird die Kernfrage der historischen Epistemologie – wie wissenschaftliche Diskurse in der Konstellation bestimmter historischer Kontexte zustande kommen (Daston 1994) – direkt mit den Fragen der politischen Epistemologie verbunden, welche die politischen Dimensionen und Implikationen wissenschaftlicher Diskurse aufgreifen (Kleeberg & Suter 2014; Vogelmann 2022). Die Fragen der politischen Epistemologie sollen dabei noch radikaler gestellt werden (Cain et al. 2019), da die politischen Ansichten der Wissenschaftler:innen – so die These – für die Entstehung diverser Theorien der Intelligenz eine konstituierende, nicht nur kontextualisierende Rolle spielten. Für die Veranschaulichung dieser Tendenz wird hier eine lange historische Periode abgedeckt, um das auf diese Weise neujustierte Feld einer Geschichte der Intelligenzdiskurse zunächst einmal grob zu umreißen.

Im Zentrum der Untersuchung stehen historiografisch weniger bekannte Kontexte, die hauptsächlich aus der Zeit stammen, in der sich die heute prominenten Diskurse der Intelligenz als geistige Fähigkeit eines Individuums oder als soziale Gruppe noch nicht etabliert hatten beziehungsweise im Entstehungsprozess befanden. Zugleich veranschaulichen die untersuchten Quellen die moderne Phase der Ablösung der „Intelligenz“ von den Assoziationen mit göttlichen und ewig unveränderbaren Fähigkeiten, die diesen Begriff in den vormodernen Diskursen geprägt hatten (Daston 2005: 41–45). In diesem Sinne sollen primär die unterforschten Vorstellungen von Intelligenz als kollektivem Phänomen besprochen werden, die allerdings die Grundlage dafür geschaffen haben, dass „Intelligenz“ später bei der Klassifizierung der Individuen nach bestimmten sozialen Gruppen zum Kriterium werden konnte. Während „Intelligenz“ in der jüngsten Forschung in Bezug auf die soziale Praxis der Unterscheidung ausführlich diskutiert wurde (Schregel 2025), soll sie hier als *Instrument* der sozialen Gruppierung beziehungsweise als integrierendes Element entsprechender sozialer Gebilde untersucht werden.

Im Folgenden werden drei Fallbeispiele behandelt, die aus unterschiedlichen historischen Zusammenhängen kommen. Das erste untersucht die Geschichte des Aufstiegs des Intelligenzdiskurses vor dem Hintergrund der Entstehung moderner, wirtschaftsorientierter Kommunikationstheorien. Es handelt sich um die Vorstellung, nach welcher „Intelligenz“ aus einem Netzwerk von wirtschaftsrelevanten Informationen entstand und ein offenes, anwendbares Wissen darstellte. Das zweite Fallbeispiel beschäftigt sich mit den Debatten um „Intelligenz“ als Machtstruktur, die die soziale und politische Entwicklung im Rahmen eines Staates mechanisch oder organisch leiten sollte – eine Vorstellung, die vor allem in der ersten Hälfte des 19. Jahrhunderts prominent wurde. Das dritte Fallbeispiel untersucht die um 1900 geführten soziologischen Debatten über Intelligenz als soziale Gruppe, die als Trägerin historisch akkumulierten Wissens dargestellt und in die soziologischen Theorien der Klassendifferenzierung eingebettet wurde.

In allen drei Fällen werden nicht Definitionen von „Intelligenz“, sondern ihre Funktion in Bezug auf die Plausibilisierung der jeweiligen sozialen und politischen Imaginationen untersucht. Der Schwerpunkt der Untersuchung liegt auf deutschsprachigen Intelligenzdiskursen, die allerdings als Teil allgemeinerer wissenschaftlicher Debatten besprochen werden. Da eine tiefgreifende Analyse aller relevanten Kontexte der untersuchten Diskussionen in diesem Format nicht erreichbar ist, kann in diesem Aufsatz nur eine selektive Quellenanalyse erfolgen. Die Unterschiede zwischen den Schwerpunkten einzelner Teile bestimmen auch verschiedene Akzente in der Materialdarstellung. Der erste Teil zeichnet die allgemeine Tendenz in der Entwicklung der wirtschaftsorientierten Intelligenzdiskurse nach und fokussiert hauptsächlich auf die frühneuzeitlichen Debatten, die in den politökonomischen Schriften und den „Intelligenzblättern“ geführt wurden. Der zweite Teil untersucht dagegen eine konkrete, in den politischen Schriften des frühen 19. Jahrhunderts entfaltete Polemik über die Rolle der „Intelligenz“ in der Struktur des Staates, die veranschaulichte, dass sich die mit ihr in Zusammenhang gebrachten sozialen und politischen Fragen zu dieser Zeit grundlegend änderten. Der letzte Teil legt den Fokus auf die diskursschaffenden Figuren des sozialdemokratischen Lagers, da sich um 1900 die gruppenbezogene Vorstellung von „Intelligenz“ hauptsächlich in dieser spezifischen sozialpolitischen Nische entwickelte.

Die Besprechung dieser drei Zusammenhänge erhebt nicht den Anspruch, eine erschöpfende Geschichte der Intelligenzdiskurse im untersuchten Zeitraum zu liefern. Das vorgeschlagene begriffs- und ideenhistorisch gestaltete Panorama bietet allerdings die Möglichkeit, die in der Wissenschaftsgeschichte routinisierten Vorstellungen von „Intelligenz“ und ihrer sozialpolitischen Wirkung zu revidieren. Die inneren Logiken der Verdichtung der untersuchten Intelligenzdiskurse sowie die grundsätzlichen Unterschiede zwischen ihnen werden hier als Projektion des Wandels der sozialen und politischen Imagination begriffen. Zugleich wird auf die Kontinuität der Funktion des Intelligenzdiskurses in verschiedenen Kontexten hingewiesen – nämlich auf die Zuschreibung (kollektiver) Agency zu diversen sozialen Gebilden. Damit soll ein Ansatz zur Analyse der Intelligenzdiskurse vorgestellt werden, der sich auch auf andere Quellen anwenden lässt.

## Intelligenz als Netzwerk

Im Jahr 1637 erteilte der englische König Karl I. seinen Untertanen den Auftrag, ein *Office of Intelligence* zu gründen (Beckmann 1817: 486). Eine Zeitschrift, die Jahrzehnte später die durch dieses *Office* gesammelten Informationen in Form eines Informationsblattes publizierte, erhielt einen äußert ausdrucksstarken Titel, der auf die politökonomische Theorie hinter diesem Projekt verwies: *The weekly information from the Office of Intelligence: […] Communicating intelligence to all merchants, traders, and all other persons […] for the propagation of trade, the general benefit of the nation, and the accomodation of every particular person *(Williams 1657). Die Funktion solcher Intelligenzbüros, die sich fast parallel in den meisten europäischen Ländern verbreiteten (Tantner 2015: 17–66), bestand darin, Verteilung, Steuerung und Austausch von praktisch und wirtschaftlich relevanten Informationen zu gewährleisten (z. B. Dury 1650: 16–17). Die Idee einer gezielten Organisation der Zirkulation relevanter Informationen stand in engem Zusammenhang mit der Entstehung frühneuzeitlicher Kommunikationstheorien (z. B. Darnton 2000; Sheehan & Wahrman 2015: 55–56, 183–223). In diesem Kontext erhielt „Intelligenz“ nicht nur den Status nützlichen und angewandten Wissens, sondern auch eine institutionelle Struktur, die ihre Entwicklung und Steigerung fördern sollte.

Der britische Wissenschaftler preußischer Herkunft Samuel Hartlib, der in den europäischen intellektuellen Kreisen *great Intelligencer* genannt wurde (Stimson 1940: 312), spielte in der Entwicklung frühmoderner Kommunikationstheorien eine führende Rolle (Greengrass et al. 1994: 1–27). In einem Pamphlet aus seinem Archiv wurden Intelligenzbüros als zentrales Instrument der erwünschten gesellschaftlichen Organisation konzipiert:

„This Office [of Intelligence] may be an Instrument, by being made a Common Intelligencer for All, not only of things actually offered or desired by some to be communicated, but also of things by himself and others observable, which may be an occasion to raise matter of communication for the Information to All“ (K.N. 1 1648: 5).

Auf diese Weise wurde „Intelligenz“ nicht nur zu einer Art normativ bearbeiteten Wissens, sondern auch zur Trägerin der Prinzipien von Offenheit und Nützlichkeit. Hartlib und sein *Hartlib Circle* gehörten zu den Gründungsmitgliedern des sogenannten *Invisible College*, das laut einem der Teilnehmer eine *Utopian Intelligence* darstellte (McKie 1960: 22). Im Hintergrund dieses Projekts stand die Vorstellung von einem *empire of knowledge*, das nicht nur die entscheidenden Informationen aus den Peripherien sammeln und ins Zentrum bringen, sondern auch ein komplexes und selbstregulierendes Netzwerk von Sammler:innen der Intelligenz etablieren würde (Findlen 2019: 1–22). Dabei ging es nicht nur um die Organisation von Wissen, sondern auch um die Steigerung der Effizienz wirtschaftlicher Prozesse, die von der Beteiligung zahlreicher Akteur:innen nur profitieren konnten (Schuck 2019: 5–10). Bereits in diesem Kontext wurde der Intelligenzdiskurs zum Instrument der Kommerzialisierung von Wissen sowie der sozialen und politischen Imagination im Allgemeinen.

Im deutschsprachigen Kontext wurde der kommerz- und kommunikationsbezogene Intelligenzbegriff bereits im späten 17. Jahrhundert breit rezipiert. In seinem Werk *Fürstliche Schatz- und Rentkammer*, das zu den zentralen Schriften der damaligen Politökonomie zählte und in den wirtschaftlichen Debatten des 18. Jahrhunderts immer wieder als klassisches Werk Anerkennung fand (Srbik 1910: 136–153), schrieb der Merkantilist Wilhelm von Schröder (1640–1688) im Teil „Von Verbesserung des Landes“:

„Der name dieses intelligenzwerks, wie auch die erste invention desselben an sich selbsten, ist erstlich vor wenig jahren von den Engelandern herkommen und erfunden, und deshalben zu Londen auf der börse eine öffentliche intelligenzkammer, sub nomine office of intelligence oder officium intelligentiae, sub auctoritate publica, auf- und angerichtet worden, welcher sache unvergleichlichen nutzen ich erwogen, und deshalben solches auch in diesen landen introducirt zu werden, ich es nach dem ingenio dieser länder und ihrer beschaffenheit gemäs elaboriret“ (Schröder 1752 [1686]: 355).

Schröders Gesamtkonzept trug den ausdrucksvollen Titel „Project eines freywilligen ungezwungenen intelligenzwerks zur consolation der länder, ingroßirung der commercien, propagirung der manufakturen, und vermehrung Jhro Kayserl. Majest. einkommen, nüzlich und dienlich erfunden“ (Schröder 1752 [1686]: 355). Ohne die Loyalität zum Kaiser zu hinterfragen, sah Schröder das „intelligenzwerk“ als „frei“ und „ungezwungen“, was sein politökonomisches Programm widerspiegelte: „Nützlichkeit“ und die Bereicherung des Landes wurden zu Argumenten, die die königliche Regierung davon überzeugen sollten, dass eine bestimmte Selbstregulierung innerhalb des Landes (im Rahmen des merkantilistischen Programms) zugelassen werden sollte.

Dieses Programm implizierte auch eine radikale Änderung der Vorstellungen davon, wie die Wissensproduktion organisiert werden solle und wer an dieser Produktion beteiligt sein könne. Im frühen 18. Jahrhundert wurde in einer der Weimarer Zeitungen intensiv diskutiert, wie die „Curiosität der Leute“ dazu genutzt werden könne, sie in den Prozess der Produktion allgemein nützlichen Wissens zu integrieren. Das „Intelligenzwesen“, das sich zu diesem Zeitpunkt im deutschsprachigen Kontext diskursiv und institutionell wohl etabliert hatte, wurde zum Mittel der Bekämpfung der „schädlichen Unwissenheit im gemeinen Leben“:

„Und eben deshalb ist das sogenannte Intelligenz-Wesen ein sehr nützliches Unternehmen, indem solches eben dahin gerichtet ist […], sonderlich aber den Nutzen dieser natürlichen Begierde, in welchen die Gelehrten bißhers fast ein Monopolium gehabt, allgemein zu machen, und dieselbe auf eine tugenhafftige und vernünftige Art, auch bey geringen Leuten, zu vergnügen“ (1733, cit. ex. Böning 2023 I: 9).

Dabei handelte es sich nicht nur um aufklärerische Ideen der Erziehung des „einfachen Volkes“, sondern auch darum, dass die Beteiligung an der Produktion und Rezeption „nützlichen Wissens“ und damit ein Engagement im wirtschaftlichen Leben des Landes zu einem allgemein zugänglichen Instrument der moralischen Stabilisierung werden sollten, das nicht mehr unter dem Monopol der Gelehrten bleiben durfte (Israel 2001: 252).

Im Lauf des 18. Jahrhunderts spielte die politökonomische Logik eine immer größere Rolle in wissenschaftlichen Diskursen (z. B. Schabas & De Marchi 2003). Der Intelligenzdiskurs wurde dabei zu einer der Ausdrucksformen der neuen politökonomischen Imagination, was bereits in der Mitte des 18. Jahrhunderts am routinisierten Wortgebrauch sichtbar wurde. Die erste Definition des Begriffs der *Intelligence* im 1755 publizierten Wörterbuch des bekannten englischen Lexikografen Samuel Johnson (1709–1784) lautete: „Commerce of information; notice; mutual communication; account of things distant or secret“, die zweite: „Commerce of acquaintance; terms on which men live one with another“; die dritte: „Spirit; unbodied mind“. Die aus heutiger Sicht selbstverständlich erscheinenden und auf die Fähigkeiten eines Individuums bezogenen „understanding“ und „skill“ kamen erst in den letzten Zeilen des Beitrags vor (Johnson 1755). „Kommerz der Information“ stellte nicht nur ein spezifisches Verständnis von Wissensproduktion dar, sondern schilderte auch den politökonomischen Ansatz zur Erklärung der Mechanismen der Entstehung des moralischen Konsensus als Grundlage, auf der „Menschen miteinander leben“.

Das Zusammenspiel der kommunikativen Aspekte und der Mechanismen der moralischen Stabilisierung war im deutschsprachigen Kontext nicht weniger sichtbar, da der Intelligenzdiskurs hier eine besondere Tragweite in Form von Intelligenzblättern bekam (Blome 2006; Böning 2023). Der bekannte Verleger Johann Andreas Erdmann Maschenbauer definierte „Intelligenz“ in seinem Wörterbuch *Der curiose und in allen nöthigen Wissenschafften Nützliche Dollmetscher* als „das aus der Erfahrung und Studiren herfliessende Erkäntniß“ und „Wissenschaft“, fügte aber noch „das heimliche oder gute Vernehmen“, „die Bekanntschafft“ und „Kundschafft“ hinzu. Noch entscheidender ist, dass Maschenbauer das Phänomen des „Intelligenz-Zettels“ (einer der Namen von Intelligenzblättern) in direkten Zusammenhang mit dem Begriff der Intelligenz brachte:

„Dahero [vom Begriff der Intelligenz ausgehend] gesaget wird Intelligenz-Zettel […]. Welches von dem Lateinischen geborget ist: Dann *Intelligentia popularis*, sonderlich bey dem Cicerone, dasjenige heisset, was der gemeine Mann begreiffet und verstehet […]. Heutiges Tags sind solche Blätter und Intelligenz-Zettel an vielen Orten sehr beliebt, darinn überhaupt allerhand Nachrichten in solchen Dingen bestehen, an deren zeitigen Kundschafft vielen Leuten, absonderlich in Handel und Wandel, in ihren Verrichtungen, inner- und ausserhalb Landes gelegen, und dem Publico nicht wenig Nutzen schaffen“ (Maschenbauer 1748: 326).

In seinem ausführlichen Kommentar beschreibt Maschenbauer verschiedene Arten von Informationen, die in den Intelligenzblättern ausgetauscht werden konnten. Sein zentraler Gedanke blieb allerdings sehr nah an den Ideen der früheren Netzwerktheoretiker: Durch ihre Teilnahme an der „Intelligenz“ sollten die „gemeinen Menschen“ in die wirtschaftlichen Informationsnetzwerke einbezogen werden, was wiederum zu ihrer moralischen Entwicklung beitragen konnte. Auf diese Weise wurden Intelligenzblätter zu einer gesellschaftlichen Institution, die die kommerz- und kommunikationsorientierten Intelligenzdiskurse materialisierte und zu einem greifbaren Netzwerk machte (Blome 2006: 10, 21–22).

Intelligenzblätter sollten nicht nur kommerzielle und kommunikative, sondern auch eine ganze Reihe polizeilicher und administrativer Funktionen übernehmen. „Der unbeschreibliche Nutzen des wohleingerichteten Intelligenzwesens“ (Merkel 1768: 124), wie einer der Autoren es formulierte, sollte durch die Offenheit nützlichen Wissens „ein jedes Land“ zu einem wohlorganisierten und selbstverwalteten Netzwerk machen:

„Wenn demnach in einem Staate alle Wochen, oder wenigstens alle Monate, alle und jede Personen in der Nähe und Ferne, zum Behuf ihres öconomischen Gewerbes, in eine nützliche Correspondenz und in ein freundliches Vernehmen zu treten […] wenn die sorgsame Policeydirection in tausend schönen Anstalten, neuen Gewerben, guten Erfindungen, haus- und landwirthschaftlichen Vortheilen […] alle und jede Unterthanen gleichsam öffentlich zu unterrichten, […] sich alle Mühe giebt, […] so nenne ich solches ein wohleingerichtetes und vollständiges Intelligenzwesen“ (Merkel 1768: 123–124).

Die zahlreichen Elemente, aus denen „Intelligenz“ bestand, sollten sich zu einer „wohleingerichteten“ Einheit entwickeln, die eine ständige Interaktion zwischen ihren Bestandteilen implizierte.

Die Versuche der Integration breiterer Kreise in den öffentlichen Diskurs mithilfe des „Intelligenzwesens“ nahmen im Lauf des 18. Jahrhunderts immer stärker politisch-mobilisierende Dimensionen an (z. B. Mulsow 2018: 477–484). Die Verknüpfung der Intelligenz mit der Presse brachte sie ebenfalls in Zusammenhang mit der Idee bürgerlichen Engagements. Die Teilhabe an der Intelligenz wurde zur politischen Teilhabe. In dieser Hinsicht führte das Streben nach politischer Vereinigung zu Apellen, die Lokalisierung des Intelligenzwesens zu überwinden und dadurch einen gemeinsamen bürgerlichen Raum zu schaffen. Dies war der zentrale Gedanke des „Volksaufklärers“ und Befürworters der Etablierung einer nationalen Presse Rudolph Zacharias Becker (1752–1822). Er schrieb:

„Man fühlte daher längst das Bedürfniß eines allgemeinen Intelligenzblatts für alle und jede Gegenstände des bürgerlichen Lebens [… das nationale Intelligenzblatt würde] so zu sagen, zum öffentlichen Sprachsaal […], wo Jedermann, über jeden beliebigen Gegenstand, die aller Orten zerstreuten Sachkundigen befragen, und ihre Antworten vernehmen könnte“ (1790, cit. ex Böning 2023 II: 685).

Laut Becker sollte auf diese Weise das Intelligenzwesen zur Plattform für die „gegenseitige Mittheilung der mannichfaltigen Erfahrungen, Gedanken, Wünsche und Unternehmungen der Deutschen“ werden sowie „den gemeinen Wohlstand der deutschen Bundes-Republik“ und die allgemeine „Kunst und Indüstrie aller Art“ beeinflussen (1790, cit. ex Böning 2023 II: 685).

Die „freie“ und „ungezwungene“ Intelligenz, die im Rahmen des aufklärerischen politökonomischen Projekts konstruiert werden sollte, war nicht lediglich eine Reflexion einer politischen und wirtschaftlichen Strömung. Die Rolle der Intelligenz in diesen Diskursen war entscheidend: Sie wurde zu einer bindenden Kraft, die aus sehr unterschiedlichen Akteur:innen ein politisches, moralisches und wirtschaftliches Ganzes formte. „Intelligenz“ beschrieb etwas, das verschiedene soziale Assoziationen festigte und sie zu einer (politischen) Wirkung bringen konnte. Die Entwicklung der Intelligenzbüros und der Intelligenzblätter materialisierte diese Theorien und schuf Institutionen, die zum zentralen Medium der zugrunde liegenden politökonomischen Theorien allgemeiner Beteiligung am politischen Prozess wurden.

Die integrierende politische Deutung dieses Diskurses stieß allerdings auf die Logik einer hierarchisierten Herrschaft, die nach der Französischen Revolution deutlich mehr Anerkennung erfuhr (Koselleck 1989: 13–19). Auch im preußischen Kontext fanden etatistisch orientierte politökonomische Theorien eine wissenschaftliche Legitimation (ebd.: 318–324, 586–588). In diesem Kontext wurde das Thema der „Erziehung des Volkes“ und der Bekämpfung von Vorurteilen zum Anlass, die väterliche Rolle des Staates zu unterstreichen und die Notwendigkeit umfassender staatlicher Kontrolle über das Intelligenzwesen zu propagieren (Böning 1987: 107–133). Im Lauf der Zeit entwickelte sich „Intelligenz“ somit nicht nur zu einem integrierend verstandenen Netzwerk, sondern auch zu einer staatlichen Machtstruktur.

## Intelligenz als Machtstruktur

Die Prägung der bekannten Bezeichnung Preußens als „Staat der Intelligenz“ wird häufig Hegel zugeschrieben. In seiner Antrittsvorlesung an der Universität Heidelberg sagte er: „[D]er Preußische Staat ist es, der auf Intelligenz gebaut ist“ (Gombert 1902: 309). „Intelligenz“ war kein zentraler Begriff für die hegelsche Philosophie. Allerdings fand er häufig einen Platz in den Hegelschen Werken, was die Rolle der „Intelligenz“ in den politischen und philosophischen Debatten seiner Zeit sowie in der offiziellen Ideologie des preußischen Staats unterstrich. In seiner *Philosophischen Enzyklopädie* schrieb Hegel: „Die Intelligenz fängt von der Äußerlichkeit als ihrer Bedingung, aber nicht als ihrem Prinzip an, welches sie vielmehr sich selbst ist“ (Hegel [1808–1811] 1840: 179). Eine (selbst-)reflexive Abgrenzung von der „Äußerlichkeit“ beziehungsweise der (sozialen) Umwelt wurde laut ihm zum Kriterium einer erfolgreichen Entwicklung zu einem (kollektiven) Wesen. Wie diese (kollektiven) Wesen organisiert werden, blieb eine der zentralen Fragen, die „Intelligenz“ zu einem konkreten Regierungsproblem machten.

Trotz der gebräuchlichen Zurückführung auf Hegel war die Verknüpfung zwischen „Intelligenz“ und dem (preußischen) Staat keinesfalls auf seine Werke reduzierbar. Die in den aufklärerischen, politökonomischen Schriften propagierten Tugenden des Fortschritts und der Produktionssteigerung sowie die damit verbundenen Regierungstechniken wurden zu einem der zentralen Themen der protosoziologischen Debatten des frühen 19. Jahrhunderts (Kleeberg 2012: 42–56; Stedman Jones 2006: 21–47). „Intelligenz“ wurde dabei zu einem wichtigen und historiografisch kaum beachteten Element der Plausibilisierung verschiedener Theorien der Entstehung sozialer und politischer Körper, die für eine fortschrittliche Entwicklung und die Produktionssteigerung organisiert werden mussten. Nach der Französischen Revolution wurde die Vertretung diverser sozialpolitischer Theorien bezüglich der „Natur“ des Staates zu einer besonders sensiblen Frage der politischen Positionierung im Kontext der intensivierten Propaganda der „Ordnung“ des Staates als Gegensatz zum Chaos der Revolution (Koselleck 1989).

Der Herausgeber der *Neuen Monatsschrift für Deutschland* Friedrich Buchholz (1768–1843) spielte eine wichtige Rolle sowohl in der wissenschaftlichen Rechtfertigung der preußischen Herrschaft als auch in der politischen Instrumentalisierung des Intelligenzdiskurses. Buchholz galt als Kenner der frühsoziologischen Theorien Saint-Simones und als Befürworter des positivistischen Erkenntnisideals im deutschen Kontext (Garber 2006). Die französischen frühsoziologischen Forschungserkenntnisse interpretierte Buchholz allerdings auf „preußische“ Art und Weise. In seinen Schriften entwickelte er die Theorie eines zentralisierten Staates, der als wohlorganisierter Mechanismus funktioniert und von der Staatsintelligenz gesteuert wird. In seinem frühen Buch, das unter dem Titel *Der neue Leviathan* erschien, wollte Buchholz die hobbessche Theorie erweitern und für die neuen Realitäten ständiger Entwicklung und Produktionssteigerung nutzbar machen. Er schrieb:

„[E]ine kräftige Regierung [kann] nicht gedacht werden, ohne eine Intelligenz vorauszusezen, welche der ganzen Regierungsmaschine den Impuls giebt, diese Intelligenz [kann] aber nur immer das Produkt der Einsicht aller derjenigen seyn, welche als Theile der Regierungsmaschine dem impulsirenden Staatschef am nächsten stehen“ (Buchholz 1805: 72–73).

Da im Rahmen des mechanizistischen Paradigmas die zentrale Frage der Mechanik – woher die die Bewegung treibende Energie kommt – auch in Bezug auf die sozialen und politischen Körper beantwortet werden musste (Allen 2005: 267, 271; Dyson 1997), schlug Buchholz eine Lösung für dieses Dilemma vor: Sein Ausgangspunkt war, dass die Entwicklung eines Staates von jenen Impulsen vorangetrieben wird, die die regierende Intelligenz erzeugt. Intelligenz selbst entstehe zwar aus den Interaktionen zwischen den Teilen der Regierungsmaschine, brauche aber wiederum einen Impuls, der nur vom Staatschef ausgehen kann. Buchholz’ zentraler Gedanke, dass „die Macht [in einem wohl organisierten Staat] mit der Intelligenz in einem unzertrennlichen Zusammenhange steht“ (Buchholz 1805: 35), machte sowohl „Intelligenz“ als auch Regierungsmaßnahmen insofern historisch, als sie nur auf einer konkreten Entwicklungsstufe der historischen Entwicklung „erzeugt“ werden konnten.

Im Gegensatz zu den kommerzorientierten Netzwerktheorien der Intelligenz, die eine aktive Beteiligung der unteren Schichten – oder anders gesagt: die Entstehung einer partizipativen Gesellschaft – implizierten, behauptete Buchholz’ Theorie der Intelligenz die Notwendigkeit, eine klare Hierarchie herzustellen. In dieser Hinsicht war die absolute Macht des Staatschefs die zentrale Voraussetzung, um ein stabiles Verhältnis zwischen „Concentricität und Excentricität“ zu gewährleisten. Er schrieb:

„Da, wo eine wahre Souverainetät des Staatschefs statt findet d. h. da, wo die ganze Staatsorganisation von der Beschaffenheit ist, daß die Intelligenz alles, was sie für das Beste erkennt, ohne weitere Schwierigkeiten ins Werk richten kann, da ist ein besserer Aufbau der Regierungsmaschine nach den oben angegebenen Gesezen einer socialen Mechanik nicht blos möglich, sondern sogar sehr nothwendig, wenn alles übrige Gute erhalten werden soll“ (Buchholz 1805: 63).

Die mechanischen Termini und zahlreichen Referenzen auf die newtonschen Gesetze standen bei Buchholz keinesfalls im Widerspruch zur Idee einer ständigen fortschrittlichen Entwicklung. Im Gegenteil war diese Art von Entwicklung der Punkt, an dem sich sein Leviathan von demjenigen Hobbes’ grundsätzlich unterschied. Die Intelligenz war die mechanische Triebkraft der fortschrittlichen Entwicklung, da es laut Buchholz „für die Intelligenz kein anderes Maximum giebt, als das der jedesmaligen Entwickelung in der Zeit und im Raume [… und] die menschliche Intelligenz nie als vollendet betrachtet werden kann“ (Buchholz 1805: 205).

Als Experte für die französischen Theorien bezog sich Buchholz häufig auf die postrevolutionäre politische Entwicklung Frankreichs, um Belege für seine Theorie zu finden. In einem seiner späteren Aufsätze über die Folgen der Französischen Revolution (die er für die Beseitigung des feudalen und kirchlichen Separatismus allerdings lobte) schrieb Buchholz, dass die Restauration der Monarchie in Frankreich ebenfalls eine gesetzmäßige Entwicklungsstufe darstelle. Der *Grande Terreur* und später die Herrschaft Napoleons hätten dabei geholfen, den Separatismus verschiedener Gewalten zu beseitigen. So habe das neue Regime die Möglichkeit erhalten, neue zentralisierte Machtstrukturen zu etablieren, die das Land bestmöglich regierbar machten (Buchholz 1830: 172–210). Da es sich letztlich um die Macht der Intelligenz handelte, versuchte Buchholz, aus seinen Theorien eine „positive“ Sozial- und Politikwissenschaft zu machen und behauptete, dass „die Politik, d. h. die Wissenschaft der Gesellschaft zu einer positiven Wissenschaft geworden seyn [wird], die sich aus den Banden des Conjekturalen hervorgearbeitet hat, und, wie jede andere auf Beobachtung und Erfahrung beruhende Wissenschaft, nur das gut heißt, was ihren Prinzipen gemäß ist“ (Buchholz 1830: 186). So war die zentralisierte Macht laut Buchholz insofern gut, als sie dem Fortschritt dienen konnte – das heißt auf der Intelligenz basierte.

Diese mechanizistische Organisationstheorie war für den preußischen Kontext sehr anschlussfähig. Die vermutete Organisations- und Stabilisierungskraft der Intelligenz erschien sowohl den Gelehrten als auch den Machthabern äußerst attraktiv. Prinz Wilhelm von Preußen fasste dies 1830 im folgenden Satz zusammen: „Was einst bei drei Millionen der Enthusiasmus tat, muß jetzt bei elf Millionen die geweckte und geförderte Intelligenz tun“ (zit. aus Koselleck 1989: 398f.). In diesem Kontext entstand die Idee, dass die Bürokratie zu einer quasi mechanisch organisierten Intelligenz des preußischen Staates werden konnte. Ein anonymer Autor schrieb 1832 in der *Augsburger Allgemeinen Zeitung*:

„In Preußen kennt man keinen Unterschied zwischen *Fonctionnaires* und *Employés*. Die Hierarchie der Beamten, als wirklicher Staatsdiener, ist bis zu den Untersekretairen und Registratoren hinab fortgesetzt, und in sich selbst wie eine lebendige *Staatsintelligenz* gegründet. *Die Maschine*, seit langer Zeit im Gange, erhält sich selbst, erneut sich von selbst in allen ihren Theilen, zieht sich selbst auf und bedarf nirgends der fremden Hilfe, um regelmäßig mit der Zeit fortzugehen, und die großen Stunden der Weltgeschichte anzuschlagen“ (K.N. 2 1832: 717).

Neben den Grundlagen der künftigen Organisationssoziologie und der Bürokratietheorien (z. B. Weber 1922: 650–678) wird in diesem Text deutlich, dass der Autor mit den mechanizistischen Metaphern der Staatsintelligenz der bürokratischen Maschine eine Unparteilichkeit und damit eine besondere Gerechtigkeit zuschrieb. Er behauptete, dass „Stand, Vermögen, Religion, Denkungsart, politische Gesinnung, selbst Vaterland und Nation“ für die regierende Maschine „gleichgültig“ seien (K.N. 2 1832: 717). Diese vorurteilsfreie Handlungsweise konnte der zentralisierten und hierarchisch organisierten Staatsintelligenz eine besondere moralische Autorität verleihen.

Die Theorie der Intelligenz als Instrument hierarchischer staatlicher Organisation fand scharfe Kritiker. Ein anderer anonymer Autor publizierte 1830 eine Broschüre mit dem aussagekräftigen Titel „*Über das Bedürfnis der Intelligenz unserer Zeit und die Möglichkeit, mit einer liberalen Majorität einen Staat zu regieren*“. Der Autor nannte Buchholz und seine Anhänger „Vertheidiger des politischen Obskurantismus“ (K.N. 3 1830: 4). Der Schwerpunkt seiner Argumentation lag auf der politischen und ökonomischen Initiative von unten, die Buchholz’ Wissenschaftstheorie bekämpft hatte:

„Sie [die Theorie von Buchholz] setzt voraus, dass die Staatswissenschaft sich auf die kleine Kaste ihrer Priester beschränkte und der populären Intelligenz nicht zugänglich sei […] dann dass die Wissenschaft alle Leidenschaften, allen politischen Eigennutz ihrer Bekenner ausschließe; und endlich dass diese die wahre, unumstößliche Staatswissenschaft und Weisheit wirklich besitzen“ (K.N. 3 1830: 13–14).

Der Hinweis auf den „politischen Eigennutz“ und die politische Teilhabe der „populären Intelligenz“ zeigt, dass der Autor die Fragen nach der politischen Agency, Eigeninteressen und Macht nicht losgelöst von der Frage nach dem sozialen Ursprung der „Intelligenz“ verstand.

Im Kontext der breiteren Auseinandersetzung zwischen liberalen und staatskonservativen Strömungen wurde die Theoretisierung der „Natur“ der Intelligenz zur Frage der politischen Positionierung. In diesem Zusammenhang wurde das gesamte mechanizistisch-positivistische Weltbild als Wurzel staatlichen Konservatismus und der Reaktion begriffen. „Intelligenz“ wurde stattdessen als ein organisches Phänomen dargestellt, das von den menschlichen Akteur:innen nicht trennbar war und zusammen mit der Gesellschaft eine politische und wissenschaftliche Reife erreichen musste. Menschen wurden wiederum nicht lediglich als physikalische Elemente eines Netzwerks, sondern als „natürliche“, nach Freiheit strebende Wesen begriffen, was dem frühliberalen Ideal entsprach (Skinner 1998: 78–98). Das Niveau der „Intelligenz“ wurde zum Marker der „Reife“ einer Gesellschaft und ihrer Fähigkeit zu einer politischen und wirtschaftlichen Entwicklung. Der Autor schrieb weiter:

„Die politische Intelligenz von Europa begann zu einer Höhe zu steigen, von welcher aus sie anfing das Leben des Volkes zu beherrschen. Die Französische Revolution hatte die durch die zunehmende Bildung verbreiteten Ideen auf eine übereilte Weise in die Wirklichkeit einführen wollen, und war in den Fehler des politischen Idealismus verfallen. So wenig dieser liberale Idealismus eine Stabilität erlangen kann, eben so wenig konnte seitdem der politische Idealismus der absolutistischen Ultras die Billigung der intelligenteren Menschen erhalten“ (K.N. 3 1830: 17).

Trotz der offensichtlichen liberalen Bestrebung des Autors setzte das Stadium der Entwicklung der Intelligenz laut ihm auch der „zulässigen“ Freiheit klare Grenzen. Eine unvernünftige Überholung des „Naturgangs“ (wie im Falle der Französischen Revolution) und eine „naturwidrige“ Verweigerung der Entwicklung durch die unbegrenzte Macht des Monarchen waren die Extreme, vor denen die Gesellschaft geschützt werden sollte.

Diese Argumentation verlieh der „Intelligenz“ eine unantastbare Autorität, da nur sie letztlich in der Lage war, die entsprechenden sozialen Körper in die „richtige Richtung“ zu führen und eine staatsbildende Funktion zu erfüllen. In dieser Rolle verlangte die Intelligenz eine politische Stellung: „Wer sich der vorhandenen unläugbaren Intelligenz seiner Zeit nicht anschließen mag, wer sie fürchtet aus eigensüchtigen Gründen, hat seine Zeit nicht begriffen und führt die Gesellschaft einer Gefahr entgegen, deren Folgen nicht zu ermessen sind“ (ebd.: 17).

Die Tatsache, dass dieses Argument fast identisch mit dem von Buchholz ist, ist im Kontext der radikalen Unterschiede ihrer politischen Programme besonders bemerkenswert. Ganz wie Buchholz schrieb der anonyme Autor: „Die Intelligenz ist die Aufgabe der Menschheit und alles Leben arbeitet darauf hin, sie zu steigern“ (ebd.: 21–22). Allerdings wurde diese Steigerung laut ihm nur durch die Entwicklung unabhängiger „Institutionen der Gesellschaft“ möglich, die „mit den gebildeten Begriffen von Recht und Gerechtigkeit überall in Einklang“ gebracht werden sollten. Im Gegensatz zu den Ideen der Netzwerktheoretiker, die ebenfalls „Offenheit des Wissens“ predigten, impliziert diese Theorie nicht nur eine Teilnahme an der Wissensproduktion, sondern auch eine politische Teilhabe an der Staatsbildung. Nur die politische Partizipation konnte nach Ansicht des Autors eine Art Entfesselung der „Intelligenz“ mit sich bringen:

„Darum aber ist den Vertretern des Volkes die Initiative zur Gesetzgebung mit anvertraut, damit diese nicht den Bedürfnissen des Landes und den Forderungen der Intelligenz gegenüber verknöchern, sondern sich rasch und ohne gewaltsame Störungen vorwärts bewegen könne“ (K.N. 3 1830: 30).

Die Verlagerung des Schwerpunkts von mechanizistischen zu organizistischen Theorien der Intelligenz änderte nicht die Schlüsselrolle des Intelligenzdiskurses bei der Erklärung sozialer Prozesse und der Entstehung „natürlicher“ sozialer Körper mit den entsprechenden Machtverhältnissen. Franz von Baader, mit dessen Zitat dieser Aufsatz begann und der immer wieder betonte, „daß es nun in der Natur jeder Bewegung der Intelligenz liegt, sich zu gemeinsamen und zu socialisieren“ (Baader 1826: 5), behauptete, dass Intelligenz die einzige organische Kraft sei, die in der Lage wäre, „soziale Körper“ zu ihrer Vollkommenheit zu bringen und ihnen eine Agency zu verleihen. Er schrieb:

„Meiner Ueberzeugung gemäß schreibt sich nun [der] irrige Freicheitsbegriff […] von der Verkennung der sämmtliche Intelligenzen organisch zu associiren strebenden Natur der Vernunft her […]. [Vernunft] als Anlage [befindet sich] in allen intelligenten Kreaturen disseminiert, so tritt selbe doch als wirkliche […] nur da hervor, wo wir diese mehreren Menschen sich in eine sozial-organische Einheit formieren sehen, d. h. in einem socialen Leib (corps), sey es in die Einheit einer Familie, eines Stammes, eines Volkes (Zunge) oder in die mehrerer (aller) Völker als einer Gemeinde (oder Kirche)“ (Baader 1826: 4, 6–7).

So bestand die Funktion der Intelligenz laut Baader darin, Individuen zu den sich „organisch“ und „natürlich“ entwickelnden, von der Vernunft vorgeschriebenen Formen zu bringen – ein Prozess, den er als Sozialisierung bezeichnete.

Der Diskurs des „organischen“ Regierens durch Intelligenz konnte noch offenere antiliberale Formen annehmen. Neben seinen bekannten Beiträgen zu kraniometrischen und rassistischen Theorien mentaler Fähigkeiten (Müller-Tamm 1995: 115–137) entwickelte der führende romantische Physiologe Carl Gustav Carus auch die Vorstellung von Intelligenz als sozialer und politischer Machtstruktur (I. Kleeberg 2014: 217–258). In seinen Schriften über „die Entwicklung der Volksmassen zu intelligenten Institutionen des Staats“ (Carus 1851: 177) zeigte er, wie hierarchische Strukturen „natürlich“ entstehen konnten. In einer nicht publizierten Handschrift „Vom Urbilde des Staates, eine Abhandlung zur Physiologie der Menschheit“ erarbeitete Carus seine organizistische Theorie des Staates, wobei die Intelligenz zum zentralen Element seines Regierungsmodells wurde. Sehr ähnlich wie Buchholz lobte Carus „Centricität“ als Hauptprinzip der Staatsorganisation, doch anstatt „sozialer Mechanik“ schrieb Carus vom „organischen Wachsen“ der Machtstrukturen. Im Staat von Carus steuerten die „Organe der Intelligenz“ die von sämtlichen Wissenschaften akkumulierten Erkenntnisse, was dem „Kopf“ des Staates erlaubte, die innere Stabilität zu gewährleisten. So stellte Carus den Staat als „lebendigen Körper“ beziehungsweise als Organismus mit „vegetativen und animalen Functionen“ dar, während die wichtigste Aufgabe der Intelligenz darin bestand (im Gegensatz zu Buchholz, der nach Mechanismen der Energieerzeugung suchte), sich an die Umwelt anzupassen und die aus kollektiven Perzeptionen erworbenen Erkenntnissen zu verarbeiten (Carus [1847?]: 9v).

Die Machtverhältnisse, die mithilfe des Intelligenzdiskurses legitimiert wurden, konnten sehr unterschiedlich konzipiert sein. Auf Intelligenz berief man sich, wenn man die Autorität der „Natur“ für ein bestimmtes Programm sozialer und politischer Entwicklung gewinnen wollte. Diese Programme konstruierten sehr unterschiedliche Arten von Akteuren.

Bereits an den untersuchten Beispielen werden wenigstens drei unterschiedliche Modelle von Agency erkennbar. Im ersten Fall war der Staat der einzige Akteur, der durch Intelligenz zu fortschrittlicher Entwicklung befähigt wurde. Im zweiten ging es darum, dass „das Volk“ durch Intelligenz seine Agency erkämpfen musste, um den Staat „organischer“ zu machen. Der dritte setzte auf politische Hierarchien, die aus dem „Naturgang“ erwachsen.

In den diesen Positionen entsprechenden politischen Theorien (z. B. Beiser 1996) war „Intelligenz“ ein bisher kaum beachtetes, aber zentrales Element, das den jeweiligen sozialen Körpern eine Agency verleihen konnte. Welche sozialen oder politischen Körper eine „eigene“ Intelligenz besaßen beziehungsweise von einer Intelligenz regiert wurden, war eine Frage, die nur in entsprechenden Kontexten beantwortet werden konnte. Dadurch wurde „Intelligenz“ nicht nur zu einem sich ständig entwickelnden Bindeglied verschiedener imaginierter Konstruktionen, sondern auch zu einem auf die Natur bezogenen Argument bei der Durchsetzung diverser Machtinstitutionen. Die Diskussion über „Intelligenz“ wurde zu einem wichtigen Raum politischer Positionierung.

## Intelligenz als Gruppe

Um 1900 änderten sich die sozialwissenschaftlichen Fragestellungen erheblich. Die Vorstellungen sowohl von den Funktionen als auch von den Träger:innen der Intelligenz mussten entsprechend angepasst werden. Das Wissen über die industrielle Produktionssteigerung und die Mechanismen des Kapitalerwerbs hatte sich stark spezialisiert. Die entstehenden Sozial- und Politikwissenschaften begannen, sich institutionell zu etablieren und eine neue Rolle in der Stabilisierung der politischen Struktur existierender Staaten zu spielen (z. B. Zimmerman 2016). Soziale Schichtung, Klassenbildung und Entstehung sozialer Gruppen in einer industrialisierten Gesellschaft wurden zum Schwerpunkt diverser Wissenschaften, die sich mit soziologischen Fragestellungen befassten, wobei die frühere Auseinandersetzung mit abstrakten Mechanismen der Entwicklung „sozialer Körper“ an Geltung verlor. Angesichts der Differenzierung der Vorstellungen von der politischen Struktur eines Staates und einer Gesellschaft war „Intelligenz“ nicht mehr lediglich im Sinne abstrakter sozialer Gebilde definierbar. An die Stelle der etablierten Vorstellungen vom Staat oder Volk musste der Träger der „Intelligenz“ gesellschaftlich spezialisiert und im Sinne einer sozialen Gruppe beschrieben werden. Im Hintergrund der Verbreitung der zu dieser Zeit entstehenden individualpsychologischen Intelligenztheorien (Schregel 2025: 94–136) entwickelte sich diese Ausrichtung des Intelligenzdiskurses hauptsächlich in einer spezifischen sozialpolitischen Nische, die in den meisten Fällen mit Macht- und Kapitalismuskritik assoziiert war.

Besonders prominent kann diese Tendenz am Beispiel der sozialdemokratischen Diskussionen über die emanzipatorischen Aspekte des Wissens veranschaulicht werden. Obwohl der aus dem Polnischen stammende Begriff der „Intelligenzija“ durch die publizistischen Aktivitäten der aus dem Russländischen Reich^1^ geflohenen Sozialist:innen im späten 19. Jahrhundert eine neue internationale Tragweite bekam (Geiger 1956: 302–304), war „Intelligenz“ im Sinne einer sozialen Gruppe im deutschsprachigen Kontext kein Fremdwort. Wie aus den oben dargestellten Beispielen ersichtlich wird, war die gruppenbezogene Beschreibung der Intelligenz eine logische Fortsetzung der früheren Debatten über ihre sozialen Funktionen. Dadurch, dass der Interessenkonflikt zwischen diversen sozialen Gruppen ins Zentrum der soziologischen Reflexionen rückte, musste „Intelligenz“ in Bezug auf die inneren politischen und wirtschaftlichen Verhältnisse gedeutet werden.

In seinem berühmten Aufsatz *Die Intelligenz und die Sozialdemokratie* beschrieb Karl Kautsky die Entstehung der Intelligenz als „soziale Klasse“ im engen Zusammenhang mit der Etablierung der Strukturen der kapitalistischen Gesellschaft:

„Die kapitalistische Produktionsweise setzt an Stelle des Handwerks die Großindustrie, sie zerlegt die Arbeit des Handwerks in körperliche und geistige Arbeiten und macht neben den Arbeitern an der Maschine Ingenieure, Chemiker, Betriebsleiter u. s. w. nötig. […] Auf diese und manche andere Weise fördert die kapitalistische Produktionsweise […] das Anwachsen der Intelligenz“ (Kautsky 1895).

Die „künstliche“ Trennung zwischen körperlicher und geistiger Arbeit war laut Kautsky die Auswirkung des Kapitalismus, welche zur Formierung der modernen Intelligenz führte. Das „Anwachsen der Intelligenz“ resultierte in ihrer Ansammlung in bestimmten Segmenten der Gesellschaft, wobei ihre Träger:innen nicht ohne Weiteres in die Klassenstruktur der Gesellschaft eingeschrieben werden konnten. Kautsky betonte, dass die Intelligenz aufgrund der starken Interessenunterschiede ihrer Mitglieder nicht in der Lage war, ein eigenes Klassenbewusstsein zu entwickeln. In dieser Hinsicht hatte „Intelligenz“ die Funktion, verschiedene gesellschaftliche Elemente zu binden. Wer mit wem – und gegen wen – durch Intelligenz gebündelt wurde, war eine wirtschaftlich-politische Frage.

Laut Kautsky sollte die Intelligenz für die Sozialdemokratie gewonnen werden, da ihre reflexive Kraft das Proletariat stärkte und die nicht abwendbare Entwicklung des historischen Prozesses beschleunigen konnte. Das „Gewinnen“ war nötig, da die objektive ökonomische Notwendigkeit zur Hinterfragung des kapitalistischen Regimes und der Emanzipation des Proletariats fehlte: Die Intelligenz profitierte von der Überproduktion an geistigen Gütern im Kapitalismus und wurde zu einer Gruppe, in der die weniger erfolgreichen Vertreter:innen der herrschenden Klassen „untergebracht“ werden konnten. In dieser gesellschaftlichen Konfiguration war „Intelligenz“ ein verborgenes Instrument des Klassenkampfes, das unter unterschiedlichen Umständen sowohl der Emanzipation als auch der Unterdrückung dienen konnte: „Die größere Intelligenz bedeutet da nicht die größere Fähigkeit, das was ist, zu erkennen, sondern die größere Fähigkeit, das was man braucht und wünscht, zu verteidigen, zu rechtfertigen, als notwendig zu erweisen“ (ebd.).

Aus dieser Perspektive heraus ging es am Ende darum, eine gesellschaftliche Gruppe zu finden, die zur Eigentümerin des über Jahrhunderte akkumulierten Wissens wurde. Außerdem war „Intelligenz“ im sozialdemokratischen Kontext nicht mehr unbedingter Wegweiser und Garant einer „richtigen“ Entwicklung. Der Hinweis auf die Legitimierungskraft der Intelligenz, die sich leicht instrumentalisieren lässt, hinterfragte ihre moralische Autorität und begriff sie als reinen Stabilisierungsfaktor für bestimmte politische und soziale Verhältnisse.

Die Gefährdung der Stabilität wurde zum zentralen Vorwurf, der gegen die sozialdemokratische Agenda erhoben wurde. So widmete beispielsweise das offizielle Presseorgan des Schweizerischen Techniker-Verbandes, das sich mit sozialen, politischen und ingenieurtechnischen Fragen befasste, einen ausführlichen redaktionellen Aufsatz dem Thema des durch die sozialdemokratische Propaganda steigenden Antagonismus zwischen Kopf- und Handarbeit und beklagte, dass „der Handarbeiter die Kopfarbeit […] nicht mehr zu schätzen [weiß]“ und die Opposition zwischen ihnen „im Klassenkampfe ohne jeden Gewissensskrupel angewendet [wird]“ (K.N. 4 1910: 66). Laut dieser Zeitung waren die Entwicklung der „Intelligenz“ sowie ihre Verteilung unter den Menschen gesellschaftliche Faktoren, die aus der langen Geschichte der Menschheit hervorgingen und am Ende zur Bedingung der gesellschaftlichen Stabilität und der fortschreitenden Steigerung der Lebensqualität wurden: „[J]e mehr durch das Zusammenwohnen der Menschen sich das Bedürfnis nach qualitativ besserer Lebenshaltung steigert, desto mehr entwickelt und vertieft sich die Intelligenz“ (ebd.: 66). Dieser Logik folgend sollte die moralische Autorität der Intelligenz zurückerobert werden, indem sie zu einem Grundstein des gemeinnützlichen Haushalts wurde:

„Diese Intelligenz braucht aber Werkzeuge zu ihrer Arbeit, und dieses Werkzeug ist die Hand. Demnach gehören Kopf und Hand zusammen. […] Ein Antagonismus kann […] nicht bestehen, sondern nur ein Widerstreit zwischen den Intellektuellen und Manuellen, oder einfacher gesagt, zwischen Kopfarbeitern und Handarbeitern“ (K.N. 4 1910: 66).

„Intelligenz“ war demzufolge nicht nur in bestimmten Berufsgruppen verkörpert, sondern hatte auch Anspruch darauf, die Gesellschaft zu regulieren. Die Frage nach der Intelligenz war gleichzeitig die Frage nach der politischen Agency im Sinne der Fähigkeit eines autonomen Handelns und Regierens.

Eine wichtige Rolle spielte der Intelligenzdiskurs auch in kultursoziologischen Theorien. Im Kontext des Aufstiegs des Nationalismus, der sich auf „nationale“ Kultur und Sprache berief, wurde „Intelligenz“ nicht nur im Sinne der Trägerin anwendbarer Eigenschaften für die kapitalistische Produktion, sondern auch im Sinne einer „Kulturträgerin“ diskutiert. Diese Perspektive fügte den rein ökonomischen soziologischen Reflexionen einen wichtigen Aspekt hinzu. „Intelligenz“ als treibende Kraft des Nationalismus gefährdete die politische Ordnung der europäischen Monarchien (z. B. Trencsényi et al. 2016: 277–317). Dies implizierte aber nicht, dass die kapitalistischen Strukturen durch „Intelligenz“ hinterfragt werden konnten (ebd.: 356–390). Mit diesem Thema befasste sich der sozialdemokratische Politökonom Otto Bauer. Laut ihm stand die Intelligenz „ausserhalb des Produktionsprozesses“, wodurch „die Gegensätze und Kämpfe der Unternehmer und der Arbeiter, der Kapitalisten und der Handwerker, der Agrarier und der Industriellen sie [die Intelligenz] nicht [kümmern]“, weil „sie sie [die Kämpfe und Gegensätze] nicht versteht“ (Bauer 1907: 255).

„Intelligenz“ wurde laut Bauer jahrhundertelang in verschiedenen Sprachen gesammelt und kultiviert. Ihre Träger:innen waren aber auf die rapiden ökonomischen Änderungen der Zeit nicht vorbereitet. Mit der Entstehung der modernen Medien war die Intelligenz als soziale Gruppe imstande, vor allem das „Nationalgefühl“ durch „die Volksschule, die Zeitung, den Vortrag, das Buch“ in der Masse im Sinne einer „Volksaufklärung“ zu verbreiten. Den befreienden Kampf des Proletariats anzuführen, war sie laut Bauer nicht gerüstet (ebd.: 126). Er schrieb, dass „die Intelligenz als Klasse […] die politische Führung [verliert], sobald nicht mehr Nation gegen Nation, sondern innerhalb jedes Volkes Klasse gegen Klasse kämpft“ (ebd.: 336). So brauchte die Intelligenz seiner Meinung nach unbedingt eine Nation als sozialen Körper, um sich im politischen Kontext entfalten zu können.

Bauers Hoffnung bestand allerdings darin, dass sich die soziale Funktion der Intelligenz unter dem Einfluss sozialer Transformationen ändern würde und ihre (teilweise emanzipatorischen) Kräfte, die bisher nur im nationalen Kontext genutzt werden konnten, auch im Befreiungskampf des Proletariats eine wichtige Rolle spielen würden. Er schrieb:

„[W]enn die Intelligenz den realen wirtschaftlichen Sorgen ohne jedes Verständnis gegenübertritt, so ist sie dagegen, dank ihrer Bildung, stärker als alle anderen Klassen der Herrschaft der Ideen ihrer Zeit unterworfen. […] Darum stellt sich die Intelligenz überall an die Spitze der Kämpfenden“ (Bauer 1907: 224–225).

Auf diese Weise verbindet Bauer sehr unterschiedliche Vorstellungen von Intelligenz als geistiges Kapital, Bildung, Fähigkeiten einer Person und sozialer Gruppe, um sie als Agentin globaler politischer und sozialer Prozesse darzustellen. Nur als soziale Gruppe könnte sie einer soziologischen Analyse unterzogen werden. Was Bauer damit erreichen wollte, war eine politische Umgruppierung der Intelligenz und dadurch ihr Einsatz für die „richtigen“ politischen Zwecke.

Die Fragen der politischen Organisation der Intelligenz wurden ausführlich vom Soziologen Robert Michels (1876–1936) untersucht. Im Lauf seiner Kariere entwickelte sich Michels von einem Feministen und Sozialdemokraten zu einem von Mussolini geschätzten Experten für faschistische Kooperationstheorie (Bonnell 2023: 23). Reflexionen über die Führungsfähigkeiten der Intelligenz begleiteten ihn auf allen Stadien dieser Entwicklung (ebd.: 186–205). Sein Schwerpunkt lag allerdings auf der Gewinnung der Intelligenz für ihr politisches Engagement im modernen Parteiwesen. Eine politische Partei war laut ihm die Struktur, die das Politische zu organisieren und zu lenken vermag. Das im sozialdemokratischen Kontext diskutierte Problem der Rekrutierung der Intelligenz für politische Zwecke sollte laut Michels auf materieller Ebene gelöst werden, da auch in den Städten, „in denen die Intelligenz sich staut“, nur „ein Paar bürgerliche Intelligenzen sich der Partei anschließen dürfen, ohne sofort aus dem Brot zu kommen“ (Michels 1906: 518).

Für Michels war „Intelligenz“ ein komplexes, vielschichtiges Phänomen, das insofern eine emanzipatorische Kraft besaß, als dass nur mit ihrer Hilfe das Interesse einer Klasse zu einem politischen Programm werden konnte. Er behauptete, dass Lohnsteigerungen nicht lediglich mit dem fortschreitenden Bedarf an neuen Arbeitskräften zusammenhingen. Laut ihm geschah es hauptsächlich „dank der wachsenden Intelligenz der Arbeiter […], die die meisten von ihnen instand setzt, all das zu erringen, was der Unternehmerprofit abgeben kann“ (ebd.: 644). Auf diese Weise führe das Wachstum der Intelligenz nicht nur zu einer Kompetenzsteigerung der Arbeiter:innen, sondern auch zur Entwicklung des Klassenbewusstseins. In dieser Hinsicht war die Argumentation von Michels nicht nur wirtschaftlich, sondern auch sozialpsychologisch. Er schrieb:

„Jeder, der der Geschichte der internationalen Arbeiterbewegung auch nur einigermaßen erkenntnisbeflissen gefolgt ist, weiß, welche Summe von Arbeitslust und Arbeitsfähigkeit gerade in dem Teile des Proletariats vorhanden ist, welcher von seinem Klassenbewußtsein durchdrungen, den Entschluß gefaßt hat, sich seine Emanzipation zu erkämpfen; der weiß auch, wieviel Intelligenz, Pflichtstreue und ruhige, stetige Arbeitskraft proletarischerseits in allen Ländern entwickelt wird“ (Michels 1911: 317).

Ganz im Sinne der Agenda der Politökonomie seiner Zeit beschäftigte sich Michels schlussendlich damit, wie „Arbeitslust“, Produktivität und dadurch die Stabilität des Staates gefördert werden kann. Eine Partei, welche die Interessen der Arbeiterschaft professionell vertreten und dadurch die Zufriedenheit der Arbeiter:innen steigern könnte, war laut ihm die einzige politische Form, die zur Trägerin der spezifisch proletarischen Intelligenz werden könne.

Das Paradox der kapitalistischen Gesellschaften bestand laut Michels darin, dass einerseits „die heutige […] Wirtschaftsordnung“ „Nebenbedingungen“ dafür schuf, dass Arbeiter:innen ihre Intelligenz entwickeln, anderseits „die Monopolisierung der höchsten Kulturgüter“ durch die herrschenden Klassen dazu führte, dass „nur eine ganz verschwindende Anzahl von Lohnarbeitern ihre Intelligenz zum Intellekt durchzubilden Muße und Gelegenheit finden“ (ebd.: 317). Diese Unterscheidung zwischen der Intelligenz, die ein kollektives Phänomen darstellte, und dem Intellekt, der das Wissen zum Eigentum eines/r konkreten Arbeiters:in machen konnte, war ein wichtiger Aspekt von Michels Logik. Die Intelligenz musste laut ihm in eine Partei umgruppiert werden, um einer sozialen Klasse politische Agency zu verleihen, sodass die einzelnen Mitglieder dieser Klasse davon profitieren konnten. Ohne eine bestimmte politische Gruppierung war „Intelligenz“ wirkungslos.

Die behaupteten Organisationsformen der Intelligenz reflektierten die Vielfalt der soziologischen Ansätze zur Beschreibung sozialer Zusammenhänge: Rein wirtschaftliche, kulturalistische und politisch-organisatorische Theorien verliehen der Intelligenz sehr unterschiedliche Kompetenzen und Aufgaben. Die Gemeinsamkeit dieser Theorien war die Tendenz, Intelligenz in einer imaginierten sozialen Gruppe zu situieren, um sie soziologisch greifbar zu machen. „Intelligenz“ wurde nicht mehr mit einer unbedingt emanzipatorischen Kraft assoziiert, implizierte aber eine Fähigkeit, Gruppeninteressen zu vertreten. Für die soziologischen beziehungsweise sozialkritischen Theorien war die Zuschreibung der Intelligenz zu einer Gruppe der einzige Weg, das Phänomen der Intelligenz zu plausibilisieren. Trotz der Tatsache, dass „Intelligenz“ sich nur schwer als einheitliche Gruppe beschreiben ließ, musste sie an die neuen Vorstellungen sozialer Strukturen angepasst werden. Auf diese Weise wurde sie zu einer sozialen Kraft, die den objektiv gegebenen sozialen Prozessen eine besondere Schärfe verlieh, indem sie die Entwicklung eines Gruppenbewusstseins beschleunigen oder bremsen konnte.

## Fazit

Die dargestellten Beispiele der Auseinandersetzung mit „Intelligenz“ in verschiedenen sozialen und politischen Theorien sind weit davon entfernt, das gesamte Spektrum der Verwendung des Intelligenzdiskurses zu schildern. Die untersuchten Diskurse veranschaulichen allerdings die zentrale These, dass „Intelligenz“ eng mit der Entwicklung sozialer und politischer Imaginationen der modernen Gesellschaften verbunden war und eine wichtige, bisher kaum beachtete Rolle in der Plausibilisierung moderner sozialer beziehungsweise soziologischer Theorien spielte. Fast im Sinne des kuhnschen Paradigmenwechsels führte die Veränderung der allgemeinen Vorstellungen von sozialen Strukturen und der Entwicklung sozialer Gebilde dazu, dass die bisherigen Theorien der Intelligenz ihre Gültigkeit verloren. „Intelligenz“ musste immer wieder an die Anforderungen der entsprechenden Theorien angepasst werden. Dennoch bestand eine wichtige Kontinuität zwischen den verschiedenen Intelligenzdiskursen. In sehr unterschiedlichen Theorien erfüllte „Intelligenz“ die Funktion der Verleihung oder Verstärkung politischer oder sozialer Agency. Dies brachte „Intelligenz“ in direkte Verbindung mit der Frage danach, wer beziehungsweise welche soziale Entität als Trägerin der Intelligenz bezeichnet werden konnte und wem dadurch die politische oder soziale Agency verliehen oder zugeschrieben wurde.

Mit dieser Tendenz hing ein anderer wichtiger Aspekt zusammen: Die Entwicklung zu einem (kollektiven) selbstreflexiven Akteur implizierte eine Abgrenzung von der Umgebung beziehungsweise der Umwelt (Benson 2020; Sprenger 2021). Die Zuschreibung von Agency an Entitäten wie das nationale wirtschaftliche Netzwerk, die staatlichen Regierungsinstitutionen oder eine soziale Gruppe implizierte, dass alles, was zu diesen sozialen Körpern nicht gehörte, zu ihrer „Umwelt“ wurde. Der Charakter der Auseinandersetzung mit dieser Umwelt konnte wiederum durch die „Interessen“ der im Hintergrund stehenden sozialen Gebilde bestimmt werden, was ein weiteres Untersuchungsfeld eröffnet.

Nachdem die Relevanz der in diesem Aufsatz untersuchten Diskurse nachgelassen hatte, etablierte sich „Intelligenz“ zunächst in den Feldern der differenziellen Psychologie, sozialpsychologischen Analysen der Gesellschaftsentwicklung (z. B. Mannheim [1929] 1985, 2022) und später auch im digitalen Bereich (z. B. Seising 2021; Echterhölter et al. 2023). In all diesen Kontexten wurde „Intelligenz“ zu einer „assoziierenden Kraft“ in dem Sinne, dass sie die soziale Realität auf eine bestimmte Art und Weise „organisierte“. Der Intelligenzdiskurs implizierte, dass es eine/n Träger:in der entsprechenden „Intelligenz“ gab sowie eine Umwelt, mit der sich diese auseinandersetzte. Sowohl kollektive Entitäten als auch Individuen, die wiederum eine bestimmte soziale, professionelle, Gender- oder Altersgruppe repräsentieren, können als Träger:innen einer spezifischen Intelligenz dargestellt werden und entsprechende Umweltkonzepte mit sich bringen. Der universalistische Anspruch des Diskurses über die „künstliche Intelligenz“ (der auf mehreren Ebenen mit den im Unterkapitel „Intelligenz als Netzwerk“ besprochenen Theorien vergleichbar ist) kann ebenfalls nicht nur als Teil der Entwicklung größerer sozial-politischer Theorien (Pasquinelli 2023), sondern auch im Sinne von Agency, Träger:innen und Umwelt(en) untersucht werden.

Diese Themen, die eine weitere Erforschung verlangen, können nur aufgrund der Analyse der längeren historischen Zeiträume diskutiert werden. Die Spezialisierung der „Intelligenz“ führte allerdings zur Spezialisierung ihrer Historiografie, wobei Gemeinsamkeiten zwischen verschiedenen Intelligenzdiskursen häufig verloren gingen. Der hier verfolgte Ansatz zur Analyse der Intelligenzdiskurse, der danach fragt, welche imaginierten politischen oder sozialen Strukturen die entsprechenden Intelligenzdiskurse beglaubigen und welchen sozialen Entitäten damit eine Agency zugesprochen wird, kann auch auf andere Intelligenzdiskurse angewandt werden. Er macht sichtbar, dass die Art, über „Intelligenz“ zu schreiben, vieles über die politischen Werte und Ideale sowie über die Neigung zur Individualisierung oder Kollektivierung der Vorstellungen von sozialer Realität in verschiedenen Epochen aussagt. Angesichts der aktuell steigenden Popularität des Konzepts der „Intelligenz“ sowohl im naturwissenschaftlichen Bereich^2^ als auch in öffentlichen Diskursen kann eine solche wissenschaftshistorische Auseinandersetzung einen wichtigen Beitrag auch für öffentliche Diskussionen leisten.

## Data Availability

Alle dieser Arbeit zugrunde liegenden Daten sind in diesem Artikel enthalten.

## Footnotes

1 Dieser Begriff wird immer mehr genutzt, um die Pluralität diverser Bevölkerungsgruppen des Romanow-Reiches zu betonen (siehe z. B. Vulpius 2020).

2 Immer mehr neue Forschungsinstitutionen stellen sich zum Ziel, die Mechanismen einer kollektiven Intelligenz zu erforschen. Siehe z. B. das Projekt „Kinds of Intelligence“, (URL): http://lcfi.ac.uk/projects/kinds-of-intelligence/kinds-intelligence/; das Projekt „Science of Intelligence“ (URL): https://www.scienceofintelligence.de/; das Max Planck Institut für biologische Intelligenz (URL): https://www.bi.mpg.de/en.
